# Potential Toxic elements in shellfish from three rivers in Niger Delta, Nigeria: bioaccumulation, dietary intake, and human health risk assessment

**DOI:** 10.5620/eaht.2023011

**Published:** 2023-06-12

**Authors:** Blessing Minaopunye Onyegeme-Okerenta, Levi Okeinaye West

**Affiliations:** 1 Department of Biochemistry, Faculty of Science, University of Port Harcourt, Rivers State, Nigeria

**Keywords:** Shellfish, Potential toxic elements, Hazard Index, Bioaccumulation, Cancer Risk, Pollution Index

## Abstract

Human health risks associated with the consumption of three shellfish (*Penaeus monodon, Crassostrea rhizophorae*, and *Tympanostomus fuscatus*) harvested from the Buguma, Krakrama, and Bonny Rivers in the Niger Delta region were evaluated in this study. The bioaccumulation of potential toxic elements (PTEs) [Arsenic (As), Cadmium (Cd), Chromium (Cr), Lead (Pb), and Nickel (Ni)] was analyzed using atomic absorption spectrophotometry (AAS). The pollution index (PPI), estimated daily intake (EDI), target hazard quotient (THQ), total hazard index (HI), target cancer risk (CR), and total cancer risk (TCR) were evaluated for potential human health risks. The bioaccumulation levels of PTEs in shellfish samples followed the order: Ni > Cr > Pb > Cd > As and were above standard recommended limits except for inorganic As levels observed in *T. fuscatus* and *P. monodon* samples from Krakrama and Bonny and *C. rhizophorae* from Bonny river. The EDI values for iAs, Cr, and Ni were lower than the tolerable daily intake (TDI), however, the EDI of Cd for children in *P. monodon* (4.6E-03 mg kg^-1^day^-1^), *T. fuscatus* (1.7E-03 mg kg^-1^day^-1^) and C. rhizophorae (1.4E-03 mg kg^-1^day^-1^) from Buguma were higher than the TDI value (8.00E-04 mg kg^-1^day^-1^). The HI values were above 1. The total cancer risk (TCR) values of all analyzed PTEs for all the shellfish species from the rivers for children and adults ranged from 8.69E-04 to 2.47E-03 and 1.86E-03 to 5.30E-03 respectively and these were higher than the priority risk level (1E-04), hence, the need to monitor shellfish consumption in the study area.

## Introduction

Fish and shellfish are frequently consumed since they are affordable and easily accessible. Fishing and farming are the primary jobs and means of livelihood for the inhabitants in the coastal regions of the Niger Delta in Nigeria. Due to greater awareness of shellfish's exceptional nutritional value and acceptance of it as an important part of a healthy, wellbalanced diet, consumption of shellfish has rapidly expanded throughout the world. Iodine, lipo-soluble vitamins, low saturated fat, high-quality protein, and a variety of other crucial nutrients can all be found in shellfish [1]. Additionally, shellfish is a major dietary source of omega-3 long-chain polyunsaturated fatty acids (n-3 LC-PUFAs), particularly eicosapentaenoic (EPA, 20:5n-3) and docosahexaenoic (DHA, 22:6n-3) acids, which have been linked to several health benefits, including lowering the risk of developing cardiovascular disease, preventing certain cancers, and providing crucial nutrients for fetal growth and normal brain development in children [2]. However, because of the environmental consequences of their habitat, shellfish may be a source of potential toxic elements (PTEs), persistent organic pollutants (POPs), plastics, poisons, and parasites, which can be harmful to human health [3]. Recent anthropogenic activities, such as industrial processes and industrial effluents, domestic and agricultural use of PTEs and PTE-containing domestic wastes, and the region's predominate oil and gas exploration operations, have severely impacted the Niger Delta environment, particularly surface and ground waters, through contamination [4].

Due to their toxicity, long-term persistence, low degradation, long biological half-lives, bioconcentration, and biomagnification in the aquatic ecosystem [2, 4, 5], the ongoing release of these PTEs into the aquatic ecosystem has become a significant public health concern that poses a serious threat to humans as well as the health of the ecosystems [6]. Their ongoing presence in the aquatic ecosystem has thus raised serious public health concerns [7].

Among different aquatic organisms, periwinkle, shrimps, and oysters accumulate large quantities of PTEs due to their habitat and feeding nature. Dietary intake of contaminated food, more specifically shellfish is considered one of the major sources of total human exposure to toxic chemicals such as PTE [8].

Potential Toxic Elements of interest in this study are arsenic (As), cadmium (Cd), chromium (Cr), lead (Pb), and nickel (Ni). These PTEs are characterized by a high level of human toxicity, even at very low concentrations, and are all classified as either “known” or “probable” human carcinogens according to the International Agency for Research on Cancer [9]. Chronic intake of shellfish with these PTEs above their acceptable threshold in human beings has a multitude of deleterious effects and is associated with the etiology of some diseases, especially cardiovascular, developmental abnormalities in children, skeletal, renal, liver, neurological and neurodegenerative, bone diseases, hematologic and immunologic disorders, various types of cancer, mortality and behavioral changes [10].

Monitoring PTE levels is important due to the rise in environmental contamination brought on by illegal artisanal refineries and the bunkering of crude oil. Therefore, the goals of this study were to: (i) evaluate the bioaccumulation of potentially toxic elements such as As, Cd, Cr, Pb, and Ni in shellfish such as shrimp (*Penaeus monodon*), periwinkle (*Tympanostomus fuscatus*) and oyster (*Crassostrea rhizophorae*) from Buguma, Krakrama, and Bonny rivers (of Bonny Estuary) in the Niger Delta, Nigeria, (ii) assess the potential health hazards associated with local intake of aquatic life after exposure to PTEs, and (iii) compare the toxic element concentrations in tested shellfish species with global standards set for human consumption.

## Materials and Methods

### Study Area Description

**Figure 1.**
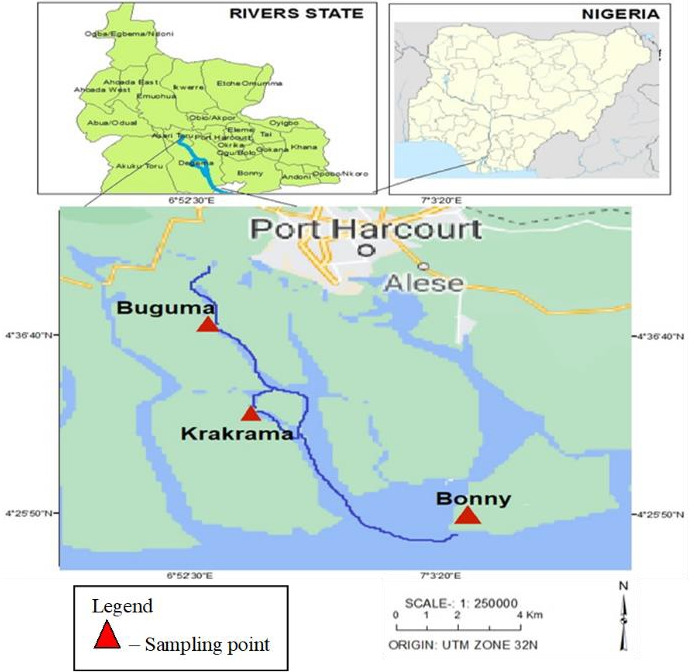
Map showing the study area [12]

Buguma River is located in Asari Toru Local Government Area of Rivers State, Nigeria. It is situated between longitude 6° 51' 44.50" E and latitude 4° 44' 10.10" N [11]. Krakrama River is located in Asari Toru Local Government Area of Rivers State, Nigeria and lies between longitudes 6°57'03.0"E latitude 4°33'04.0"N. Bonny River is located in Bonny Local Government Area of Rivers State, Nigeria. It lies between Longitude 7° 05' 60.00" E and Latitude: 4° 22' 59.99" N (Figure 1). These rivers receive a collection of waste matter from agricultural, domestic, industrial, and illegal refining activities and debris from the destruction of the illegal refiners.

### Shellfish collection, processing, and analysis

Samples were collected from the following locations: (1) George’s compound waterfront Buguma, (2) Krakama Creek in Krakama and (3) Green’s Iwoma town waterfront Bonny. For each river, three (3) selected shellfish samples, *P. monodon*, *C. rhizophorae* and *T. fuscatus*, were collected from three riverine communities in Rivers State using sterile fishing nets, knife and handpicking using hand gloves [12], and stored in an ice-packed cooler at 4°C while being transported to the laboratory before PTEs analysis. Two ml of mixed H_2_SO_4_, HNO_3_, and HClO acid in the ratio of 40%:40%:20% was added to 1 gm of the shellfish samples in a 100 ml conical flask. The mixture was digested in a fume cupboard on a hot plate at 95°C until white fumes appeared, this was allowed to cool and filtered using Whatman No. 42 filter paper into 100 ml volumetric flasks and the volume made up to 100 ml with distilled water. The filtrate was transferred into another 100 ml capacity plastic container for analysis using AAS machine model S4=71096 [13]. For every metal analyzed, atomic absorption spectroscopy (AAS) was calibrated or zeroed using a metal standard with a known concentration. Three different concentrations of the standard sample were prepared, digested, and aspirated directly into the equipment, and readings were taken. The PTEs assayed for were arsenic (As), cadmium (Cd), chromium (Cr), lead (Pb), and nickel (Ni).

### Human Health Risk Assessment

Health risk assessment is an analysis used to measure the probability of adverse health risks occurring when humans are exposed to contaminants or harmful substances [14]. In this study, the methods used by [15, 16, 17, 18, 19] were adopted in the assessment of human health risks from shellfish consumption.

The risk assessment model developed by US Environmental Protection Agency [20] was used to assess the potential harm to public health in the study area.

All children's consumption limit and health risk factors (EDI, THQ, and CR) were computed assuming 60% of adults' fish meal size of 38000 mg/day [21]. Additionally, all as consumption computations were made assuming that the toxic inorganic arsenic constituted approximately 3% of the total [19, 22, 23].

### PTE pollution index

#### Assessment of PTEs levels in sampled fish species (PTE pollution index, PPI)

The PTE Pollution Index is a mathematical model that estimates the total content of PTEs in the studied shellfish. In this study, PPI was applied to compare the total content of PTEs in the shellfish at the different sampling sites as below [16].


(1) $$\mathrm{PPI}\;\left(\mathrm{mg}\;{\mathrm{kg}}^{-1}\right)={\left({\mathrm{Cf}}_{1}\times {\mathrm{Cf}}_{2}\times \cdots \times {\mathrm{Cf}}_{k}\right)}^{1/k}$$

Where Cf_1_ is the mean concentration of the first concerned PTE, Cf_2_ is the concentration of the second concerned PTE, Cf_3_ is the concentration of the third concerned PTE, and Cf_k_ is the concentration of “kth” PTE (mg kg^−1^; ww) in the analyzed samples.

#### Estimated daily intake

Yi et al. [17] stated that the estimated daily intake (EDI) is directly influenced by the concentration of PTEs, food intake, and average body weight of the study population. The EDI is measured in mg kg^-1^ bodyweight day-1. The EDI of PTEs for the population in this study was determined by Eq. (2):


(2) $$\mathrm{EDI}=\frac{C\times \mathrm{IR}}{\mathrm{BW}}$$

The various parameters in this equation, are presented in Table 1. An assumptions was made that cooking has no impact on the pollutants and that the ingestion dose is equivalent to the adsorbed contaminant dose [19].

#### Non-carcinogenic Effects

The target hazard quotient (THQ) is an estimation of the risk level (non-carcinogenic) due to PTE exposure. To estimate the human health risk from consuming PTE-contaminated shellfish, THQ was calculated using equation (3) [18]:


(3) $$\mathrm{THQ}=\frac{C\times \mathrm{IR}\times \mathrm{EF}\times \mathrm{ED}}{\mathrm{BW}\times {\mathrm{AT}}_{n}\times {\mathrm{RfD}}_{O}}$$

Where RfD_o_ (reference dosage; mg kg^-1^day^-1^) (values used for the computation are presented in Table 3 and other parameters are presented in Table 1.

The THQ provides an indicator of the possible risk associated with exposure to particular toxicants but does not provide a quantitative calculation of the health risks for the exposed population.

The exposure of humans to two or more contaminants may result in combined or interactive effects. The hazard index (HI) is used to assess the overall potential health adverse effects posed by multiple PTEs (iAs, Cd, Cr, Pb, and Ni). It is the arithmetic sum of the individual PTE THQs, derived by Eq. (4) [27, 28].


(4) $$\mathrm{HI}=\mathrm{THQ}\left(\mathrm{As}\right)+\mathrm{THQ}\left(\mathrm{Cd}\right)+\mathrm{THQ}\left(\mathrm{Cr}\right)+\mathrm{THQ}\left(\mathrm{Pb}\right)+\mathrm{THQ}\left(\mathrm{Ni}\right)$$

Where HI is the hazard index, THQ (As) is the target hazard quotient for As intake, and so on.

If THQ or HI ≤ 1.0, indicates safe, the absence of significant non-carcinogenic health risk, whereas THQ or HI >1.0 indicates the probability of adverse health effects [21]. When HI >10, the risk is considered to be high and chronic or even acute effect [29].

#### Carcinogenic risks

According to the International Agency for Research on Cancer [9], the PTEs of As, Cd, Cr, Pb, and Ni were treated as carcinogens. Target cancer Risk (CR) is defined as the incremental probability of an individual developing any type of cancer throughout a 54.7-year lifetime of continuous exposure to a potentially carcinogenic substance. It is used to indicate or measure carcinogenic risks due to PTEs exposure. The CR was assessed by equation (5) [25]:


(5) $$\mathrm{CR}=\frac{C\times \mathrm{IR}\times \mathrm{EF}\times \mathrm{ED}\times {\mathrm{CSF}}_{O}}{\mathrm{BW}\times {\mathrm{AT}}_{c}}$$

Where CSF_o_ (mg kg^-1^ day^-1^)^-1^ is the oral cancer slope factor of each PTE studied (for As: 1.5; Cd: 3.8E-01; Cr: 0.5; Pb: 8.5E-03; Ni: 1.7) [30].

The multi-element carcinogenic risk (TCR) is the sum of the values of the single-element carcinogenic risk.


(6) $$\mathrm{TCR}=\mathrm{CR}\left(\mathrm{As}\right)+\mathrm{CR}\left(\mathrm{Cd}\right)+\mathrm{CR}\left(\mathrm{Cr}\right)+\mathrm{CR}\left(\mathrm{Pb}\right)+\mathrm{CR}\left(\mathrm{Ni}\right)$$

Where TCR is the total target cancer risk, CR (As) is the target cancer due to As intake, and so on.

If TR > 1E-04, it indicates a carcinogenic risk. If 1E-06< TR < 1E-04, it indicates an acceptable level of carcinogenic risk, and if TR < 1E-04, it indicates a negligible carcinogenic risk [27, 31].

#### Quality control

For every set of samples, a procedural blank and a standard solution consisting of all the reagents were run to check for interferences and cross-contamination. The instrument was calibrated by injection of the standard mixture at seven different concentrations to prepare the standard curve for external calibration purposes. All test batches were evaluated using an internal quality approach and validated if they satisfied the defined internal quality controls. For each experiment, a run included blank, certified reference materials (CRM) as an internal standard in samples, and samples were analyzed in duplicate to eliminate any batch-specific error. A multi-element standard solution was used to prepare a standard curve. Before starting the sequence, the relative standard deviation (RSD, <5 %) was checked by using a tuning solution purchased from Agilent company. Five standards with standard linear regression and internal standardization were prepared at levels ranging from 0 to 50 μg/L for As, Cd, Cr, Pb, and Ni. The calibration curve was plotted from six points, including the calibration blank.

#### Statistical Analysis

Statistical analyses were conducted with a one-way ANOVA test for the difference between rivers and the student t-test for the difference between shrimp, oyster, and periwinkle, with p<0.05 applied as the minimum level of significance. Statistical software SPSS 25 was used for the analyses.

## Results and Discussion

### Bioaccumulation of PTEs

**Figure 2.**
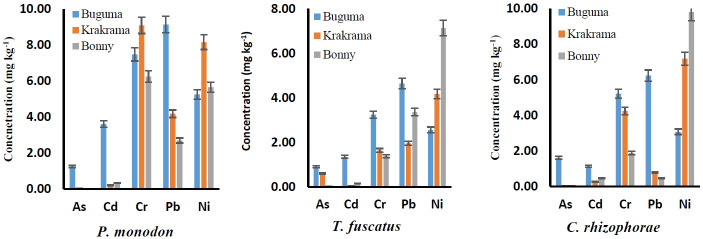
Mean concentration of As, Cd, Cr, Pb, and Ni of shellfish from the rivers

The bioaccumulation of the five PTEs (As, Cd, Cr, Pb, and Ni) in the studied shellfish samples is presented in Figure 2 by mean values and standard deviations. All the results are expressed as mg kg^-1^ dry weight. Among the shellfish species and the rivers, a wide range of PTEs bioaccumulation was observed. The mean concentrations of analyzed PTEs in *P. monodon*, *T. fuscatus*, and *C. rhizophorae* from rivers were in the following trend: Cr > Ni > Pb > Cd > As, Ni > Pb > Cr > Cd = As and Ni > Cr > Pb > Cd > As, respectively. In this present study, the highest concentrations of the components were measured in *P. monodon*. They are Cd (3.62 mg kg^-1^), Cr (9.1 mg kg^-1^), and Pb (9.15 mg kg^-1^) while the highest levels of As (1.61 mg kg^-1^) and Ni (9.81 mg kg^-1^) were measured in *C. rhizophorae*. The highest concentrations of As, Cd, and Pb were found in the shellfish from the Buguma River, whereas the highest concentrations of Cr and Ni were found in the shellfish from the Krakrama and Bonny rivers. For all PTEs, no particular variety of shellfish consistently scored high. Ni and As had the highest and lowest mean concentrations, respectively.

One of the most well-known poisons and a major threat to human health is inorganic arsenic. The richest organic sources of arsenic include shellfish, fish, and algae, which are contaminants in food, water, and the environment [32]. The *C. rhizophorae* from Buguma accumulated the highest level of As (1.61 mg kg^-1^) while a significantly low concentration of As (0.01 mg kg^-1^) was observed in *P. monodon* from Bonny. There are different forms of Arsenic compounds: metalloid (As^0^), inorganic [arsenite (As^3+^) and arsenate (As^5+^)], organic (arsenobetaine and arsenocholine), and arsine (AsH_3_) and they are classified according to their increasing toxicity in the following order: organic arsenicals < As^0^ < arsine < inorganic species (As^5+^ < As^3+^) [33]. The International Agency for Research on Cancer has classed the inorganic species of arsenic As(III) and As(V) as Class I compounds, carcinogenic to humans, and they are the most hazardous of the arsenic forms [9]. Humans are most exposed to arsenic through oral intake of contaminated shellfish, however very little is absorbed through skin contact or inhalation. Inorganic arsenic and its methylated metabolites are extremely carcinogenic, and long-term low-level exposure to them has been linked to damage or dysfunction of almost all vital organs in the human body. These include effects on vital proteins/enzymes, skin lesions, nephrotoxicity, neurotoxicity, hepatobiliary disorders, cardiovascular and peripheral vascular disease, diabetes mellitus, anemia, leukopenia, and eosinophilia [34].

The Cd concentrations in shellfish from Buguma exceeded the allowable limits of 0.5 and 0.05 mg kg^-1^ reported by the Joint FAO/WHO Expert Committee on Food Additive [35] and the European Commission, [36], respectively. The mean bioaccumulation of Cd observed in shellfish samples ranged from 0.05 to 3.62 mg kg^-1^ (Figure 2). The World Health Organization (WHO) classifies cadmium as a cell death/cell proliferation element and the International Agency for Cancer Research [9] as a carcinogenic element due to its high toxicity for humans [37]. It has a lengthy biological half-life that is suited for bioaccumulation, ranging from 17 to 30 years in humans [38]. When Cd is ingested suddenly, it can cause intestinal erosion, unconsciousness, stomach pain, a burning feeling, nausea, vomiting, salivation, lung, hepatic, or renal damage, as well as coma while chronic exposure to Cd can result in an increased risk of kidney dysfunction, brittle bones, and lung damage, prostate, pancreas, breast, and colon cancers as well as hepatocellular carcinoma in humans [37].

*Penaeus monodon* from Krakrama had the largest Cr accumulation (9.1 mg kg^-1^), whereas *T. fuscatus* from Bonny had the lowest (1.38 mg kg^-1^). These bioaccumulation values exceed the 0.5 mg kg^-1^ acceptable level for shellfish set by the European Union (EU) [38]. The International Agency for the Research of Cancer (IARC) [9] has categorized Cr(VI), one of the forms of Cr, as a group 1 human carcinogen due to its mutagenesis characteristics [37]. Exposure to Cr is connected to several ailments and extends from DNA damage, cutaneous, renal, neurological, and gastrointestinal problems to the development of many cancers including lungs, throat, bladder, kidneys, testicles, bone, and thyroid [39].

Lead is a carcinogen and a toxic substance that is detrimental to humans [40, 41]. *Penaeus monodon* from Buguma had a substantially higher Pb level (9.15 mg kg^-1^), but *C. rhizophorae* from Bonny had a significantly lower Pb content (0.47 mg kg^-1^). Lead toxicity can be either acute or long-term. Acute exposure might result in nausea, vomiting, diarrhea, stomach discomfort, hypertension, kidney malfunction, lethargy, insomnia, arthritis, hallucinations, and vertigo. Chronic exposure to lead is linked to a number of adverse health effects in humans, including neurotoxicity, nephrotoxicity, kidney damage, mental retardation, birth defects, an increased risk of lung, kidney, stomach, and bladder cancer, autism, dyslexia, weight loss, and hyperactivity [40].

The International Agency for Research on Cancer [9] and the United States Environmental Protection Agency [41] have classified nickel and its compounds as human carcinogens. Nickel is a substance that is found in the environment. The maximum concentration of Ni (9.81 mg kg^-1^) was accumulated by the *C. rhizophorae* from Bonny and Buguma, whilst the lowest Ni content (2.56 mg kg^-1^) was discovered in the *T. fuscatus* from Buguma. Ni and its compounds cause a variety of health issues through bioaccumulation in the human body, including severe pulmonary issues (cancer of the respiratory tract, lung fibrosis, emphysema, tumors), nasal cancer, epigenetic effects, gastrointestinal manifestations, kidney and cardiovascular diseases [42].

### Health risk assessment

#### PTE Pollution Index (PPI)

The total PTE contents of the shellfish species from the three rivers examined in this study were compared by calculating the PTE pollution index (PPI). The levels of contamination in the shellfish from the research regions are shown in Table 2. The following rankings were obtained from the PPI evaluation of the three rivers' shellfish species: The Buguma River contains *P. monodon* > *C. rhizophorae* > *T. fuscatus*, whereas Krakrama and Bonny Rivers contain *P. monodon* >*T. fuscatus* > *C. rhizophorae*. Additionally, the analyzed shellfish species' total PTE accumulation concentrations (represented by the computed PPI from the rivers) are distributed in the following order: Buguma > Krakrama > Bonny. While *C. rhizophorae* (mean PPI 0.95) has the lowest bioaccumulation capacity of the three shellfish species examined and analyzed, *P. monodon* (mean PPI 1.35) has the highest capacity for PTE bioaccumulation and a less active mechanism for their discharge. The conclusion is that *P. monodon* and *C. rhizophorae* are more susceptible to PTE pollution than the other species under study and can be utilized as a bio-indicator of PTE contamination. The range of PPI determined for shellfish species, 0.41–2.77, compared favorably with PPI reported in several works of literature, including 0.92–3.72 [44], 0.41–1.82 [45], and 0.25–1.15 [46], and was lower than 5.7–15.03.

#### Estimated Daily Intake (EDI)

Table 3 shows the estimated daily intake (EDI). These values were compared with the tolerable daily intake (TDI) recommended for the examined pollutants by the Joint FAO/WHO Expert Committee on Food Additive [23, 35, 47, 48]. The EDI values for iAs, Cr, and Ni for the shellfish from the three rivers were generally lower than the corresponding TDI values, indicating that the consumption of the studied shellfish species from the rivers did not have a harmful impact on consumers' health. However, the EDI values of Cd for child age class in *P. monodon* (4.6E-03 mg kg^-1^day^-1^), *T. fuscatus* (1.7E-03 mg kg^-1^day^-1^) and *C. rhizophorae* (1.4E-03 mg kg^-1^day^-1^) from Buguma were higher than the TDI value (8.00E-04 mg kg^-1^day^-1^). Similarly, the EDI values of Pb for both age classes are higher than the permissible TDI except in *C. rhizophorae* from Krakrama and Bonny, and *T. fuscatus* (1.1E-03) for adults from Krakrama. This predisposes the consumers of these shellfish to potential health risks.

#### Non-carcinogenic risk of PTEs by consuming shellfish

**Figure 3.**
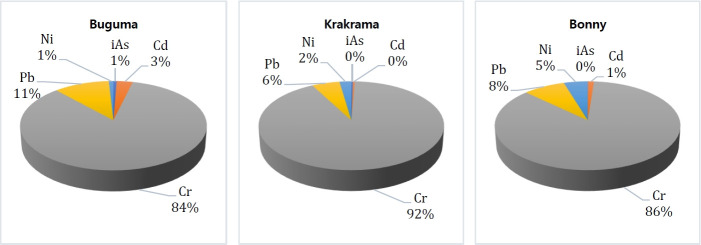
Percentage mean contribution of iAs, Cd, Cr, Pb, and Ni to Hazard risk (Hi) of shellfish from the rivers via consumption

The calculated THQ of individual PTEs for the local population on exposure through the consumption of different sampled shellfish by ingestion is presented in Table 4. According to USEPA [49], the accepted guideline value for THQ is 1. The THQ for iAs, Cd, Cr, Pb and Ni are in the following ranges: 1.21E-03 to 1.96E-01, 8.50E-02 to 1.47E+00, 5.59E+00 to 3.68E+01, 1.09E-01 to 3.70E+00, 1.55E-01 to 5.96E-01 and 5.21-04 to 8.38E-02, 8.68E-03 to 6.28E-01, 2.39E+00 to 1.58E+01, 8.16E02 to 1.08E-01, 6.66E-02 to 2.55E-01 for both child and adult age classes. The result shows that the THQ values of the iAs, Cd, and Ni in all species and Pb in *T. fuscatus* and *C. rhizophorae* from the three locations were all below 1 for both children and adults except Cd in *P. monodon* (1.78) from Buguma River for children. This indicates that the intakes of any of these single PTEs by consuming the sampled shellfish present no potential non-carcinogenic health risk except in the one abovementioned species from Buguma River. However, the THQ values of Cr in all shellfish species and that of Pb in *P. monodon* from the three rivers for both children and adults from all rivers for children were above 1, this indicates that the daily consumption of these shellfish exposed to Cr and Cd (as mentioned) are susceptible to considerable potential harmful effect on human health. In this study, chromium (Cr) was the single highest risk contributor in both children and adults which were 84%, 86%, and 92% in Buguma, Bonny, and Krakrama (Figure 3) respectively, followed by the descending order Pb > Ni > Cd > iAs.

Humans are exposed to more than one pollutant and suffer combined or interactive effects [50]. The cumulative interactive effect of all PTEs, both for children (1.13 – 5.89) and adults (3.21 – 16.8) under consideration was higher than the acceptable limit of 1 for HI in all species of shellfish. Therefore, the continuous and excessive intake of these shellfish could result in an appreciable potential adverse health risk to the human body. The highest values of HI were in the samples collected from the Krakrama River in both the children and adults, and in descending order: *P. monodon* > *C. rhizophorae* > *T. fuscatus*. The descending orders of HI values of shellfish species from the rivers were as *P. monodon*: Krakrama > Buguma > Bonny, *T. fuscatus*: Buguma > Krakrama = Bonny and *C. rhizophorae*: Buguma > Krakrama > Bonny.

#### Carcinogenic risk of PTEs via consumption of shellfish

The target cancer risk (CR) of iAs, Cd, Cr, Pb, and Ni, and the total target cancer risk (TCR) for both children and adult populations due to exposure from shellfish consumption by ingestion are presented in Table 4. The CR values of iAs, Cd, Cr, Pb, Ni ranged from 6.00E-08 to 9.65E-06, 2.53E-06 to 1.83E-04, 9.19E-05 to 6.06E-04, 5.32E-07 to 1.04E-05, 5.08E-04 to 2.22E-03 and 1.28E-07 to 2.07E-05, 5.42E-06 to 3.93E-04, 1.97E-04 to 1.30E-03,1.14E-06 to 2.22E-05,1.24E-03 to 4.76E-03 for children and adults respectively. According to USEPA [27], TCR values lower than 1E-06 are accepted as more negligible, cancer risks greater than 10−4 are unacceptable, and the range from 1E-04 to 1E-06 is assessed as acceptable. The CR values of iAs and Pb are below the priority level of 1E-04 for both children and adults and are either negligible or acceptable. The CR values of Ni (children and adults) and Cr (children and adults) were several times higher than the acceptable risk limit (1E-04) except for Cr (children) in *T. fuscatus* from Bonny River. The CR values of Cd for children and adults were within the acceptable range of 1E-06 to 1E-04, except in three cases where values were higher than the unacceptable risk (1E-04). In addition, in all species, the total cancer risk (TCR) was several times higher than the acceptable risk limit (1E-04), indicating that the people consuming these shellfish species are exposed to these PTEs with a high probability of contracting cancer risk.

**Figure 4.**
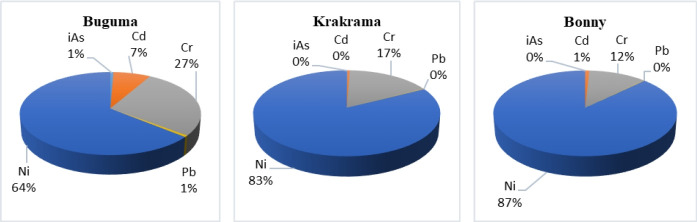
Percentage mean contribution of iAs, Cd, Cr, Pb, and Ni to total cancer risk (TCR) of shellfish from the rivers via consumption

The calculated total CR (TCR) values ranged from 1.31E-04 to 3.57E-04 (2 to 4 in 10,000) and 1.86E-04 to 5.8E-03 (2 to 60 in 10,000) for children and adults and are very unacceptable. Nickel contributed 64% in Buguma, 83% in Krakama, and 87% in Bonny while chromium contributed 12% in Bonny, 17% in Krakama, and 27% in Buguma (Figure 4). This implies that there is a cancer risk from single Ni and Cr (for both age groups) and combined PTEs exposures. The TCR of PTEs due to the consumption of shellfish through ingestion is in the descending order Ni > Cr > iAs > Cd > Pb. The TCR values for both children and adults are within 1E-03 which is higher than the priority 1E-04. It should be noted that people might be exposed to PTEs through other dietary and non-dietary routes, and thus, the real risk scenario would be greater than that estimated in this study.

## Conclusions

This study aimed to determine the concentration of five potentially toxic elements (PTEs) via consumption of shellfish from Buguma, Krakrama, and Bonny rivers in the Niger Delta and assess the associated human health risks using diverse health indices for adults and children. The results obtained from this study revealed that consumed *P. monodon*, *C. rhizophorae*, and *T. fuscatus* harvested from Buguma, Krakrama, and Bonny Rivers in Niger Delta had various concentrations of the selected PTEs and the level of accumulation varied among the samples and locations studied. The analyzed PTEs concentrations in all the shellfish samples were above the recommended limits of the Food and Agriculture Organization of the United Nations. The target hazard quotient estimated for As, Cd, and Pb for all sampled shellfish was found to be above the threshold of 1, except that of Cr in *P. monodon*, indicating potential non-carcinogenic health risks. Also, estimated HIs due to exposure to PTEs via consumption of all the sampled shellfish from the various locations were above the threshold value of 1. The examined shellfish was found to be unsafe for human consumption and hence have the potential to cause adverse health effects for both children and adult populations. The calculated total cancer risk (TCR) values of all analyzed PTEs for all the shellfish species from the rivers for children and adults were higher than the priority risk level (1E-04) which indicates a 1:10,000 probability of cancer developing in the population. Because of this, stakeholders and policymakers should help in crafting policies and strategies to mitigate the level of crude oil pollution and consequently in reducing PTEs contamination of shellfish. This study recommends that efforts should be made to minimize PTEs contamination in aquatic and terrestrial ecosystems to safeguard the biota and the health of their consumers as well as public education about the harmful effects of PTEs on human health and the environment.

## Figures and Tables

**Table 1.** Exposure parameters and values used for health risk estimation via consumption of shellfish

Parameter	Definition	Unit	Child	Adult	Reference
C	The concentration of each PTE in shellfish	mg/kg	Table 2		This Study
IR	Ingestion rate of shellfish	kg/capita/day	0.019	0.038	[21]
EF	Exposure frequency	Days/year	350	350	
ED	Exposure duration	years	6	30	
BW	Bodyweight	kg	15	70	[24]
AT_nc_	Averaging time for non-carcinogenic	Days/year	2190	10950	
AT_c_	Averaging time for carcinogenic	Days/year	19966	19966	[25]
RfD	Reference dose (oral)	mg/kg/day	Table 3		[26]

* Average shellfish consumption rate was set at 0.038 kg day-1 per person from the annual per capita fish consumption of 13.3 kg for Nigeria,

** Life expectancy for Nigeria is 54.7 years [25]

**Table 2.** PTE Pollution Index (PPI) (mg kg^−1^) of shellfish species sampled from the study area

River	Shellfish specie	PPI	Index Value ^*^	Degree of Pollution^*^
Buguma	*P. monodon*	4.40	2 < PPI <5	very low contamination
	*T. fuscatus*	2.16	2 < PPI <5	very low contamination
	*C. rhizophorae*	2.83	2 < PPI <5	very low contamination
	Mean	3.13	2 < PPI <5	very low contamination
Krakrama	*P. monodon*	1.21	PPI < 2	not impacted
	*T. fuscatus*	0.83	PPI < 2	not impacted
	*C. rhizophorae*	0.76	PPI < 2	not impacted
	Mean	0.93	PPI < 2	not impacted
Bonny	*P. monodon*	0.80	PPI < 2	not impacted
	*T. fuscatus*	0.70	PPI < 2	not impacted
	*C. rhizophorae*	0.66	PPI < 2	not impacted
	Mean	0.72	PPI < 2	not impacted

* Jamil et al., [43]

**Table 3.** EDI, RfD, and TDI values of PTEs in shellfish in the study area

Species	River	iAs^*^		Cd		Cr		Pb		Ni	
		Child	Adult	Child	Adult	Child	Adult	Child	Adult	Child	Adult
*P. monodon*	Buguma	4.8E-05	2.1E-06	4.6E-03	2.0E-03	9.5E-03	4.1E-03	1.2E-02	5.0E-03	6.7E-03	2.9E-03
	Krakrama	1.5E-06	6.5E-08	2.7E-04	1.1E-04	1.2E-02	4.9E-03	5.3E-03	2.3E-03	1.0E-02	4.4E-03
	Bonny	3.8E-07	1.6E-08	4.5E-04	1.9E-04	7.9E-03	1.8E-03	3.4E-03	1.5E-03	7.2E-03	3.1E-03
*T. fuscatus*	Buguma	3.4E-05	1.5E-06	1.7E-03	7.3E-04	4.1E-03	1.8E-03	5.9E-03	2.5E-03	3.2E-03	1.4E-03
	Krakrama	2.2E-05	9.6E-07	6.3E-05	2.7E-05	2.1E-03	8.9E-04	2.5E-03	1.1E-03	5.3E-03	2.3E-03
	Bonny	5.3E-06	2.3E-07	1.9E-04	8.1E-05	1.7E-03	7.5E-04	4.3E-03	1.8E-03	9.0E-03	3.9E-03
*C. rhizophorae*	Buguma	6.1E-05	2.6E-06	1.4E-03	6.1E-04	6.6E-03	2.8E-03	7.9E-03	3.4E-03	3.9E-03	1.7E-03
	Krakrama	6.8E-06	2.9E-07	3.4E-04	1.5E-04	5.4E-03	2.3E-03	1.0E-03	4.3E-04	9.1E-03	3.9E-03
	Bonny	1.1E-06	4.9E-08	6.0E-04	2.6E-04	2.4E-03	1.0E-03	6.0E-04	2.6E-04	1.2E-02	5.3E-03
RfD^a^ (mg kg^-1^day^-1^)		3.0E-04		1.0E-03		3.0E-03		3.0E-03		2.0E-02	
TDI^b^ (mg kg^-1^day^-1^)		2.14E-03		8.00E-04		3.00E-01		1.50E-03		1.20E-01	

EDI (Estimated Daily Intake, mg kg-1day-1). EDI values higher than the corresponding TDIs are in italics, TDI: tolerable daily intake, Rfd: Oral reference dose

* iAs (inorganic arsenic) are assumed to be 3% of the measured total Arsenic [22]; a [27]; b [23, 35].

**Table 4.** Target hazard quotient (THQ), Target Cancer Risk (CR) for different PTEs and their hazard index (HI), and total cancer risk (TCR)

Species	River	iAs		Cd		Cr		Pb		Ni		HI = ΣTHQ	
		Child	Adult	Child	Adult	Child	Adult	Child	Adult	Child	Adult	Child	Adult
*P. monodon*	Buguma	1.5E-01	6.6E-02	1.5E+00	6.3E-01	3.0E+01	1.3E+01	3.7E+00	1.6E+00	3.2E-01	1.4E-01	3.6E+01	1.5E+01
	Krakrama	4.9E-03	2.1E-03	8.5E-02	3.6E-02	3.7E+01	1.6E+01	1.7E+00	7.3E-01	5.0E-01	2.1E-01	3.9E+01	1.7E+01
	Bonny	1.2E-03	5.2E-04	1.4E-01	6.1E-02	2.5E+01	1.1E+01	1.1E+00	4.7E-01	3.4E-01	1.5E-01	2.7E+01	1.2E+01
*T. fuscatus*	Buguma	1.1E-01	4.7E-02	5.5E-01	2.3E-01	1.3E+01	5.6E+00	1.9E+00	8.1E-01	1.6E-01	6.7E-02	1.6E+01	6.8E+00
	Krakrama	7.2E-02	3.1E-02	2.0E-02	8.7E-03	6.6E+00	2.8E+00	7.9E-01	3.4E-01	2.5E-01	1.1E-01	7.8E+00	3.3E+00
	Bonny	4.1E-03	1.8E-03	6.1E-02	2.6E-02	5.6E+00	2.4E+00	1.4E+00	5.8E-01	4.3E-01	1.9E-01	7.4E+00	3.2E+00
*C. rhizophorae*	Buguma	2.0E-01	8.4E-02	4.6E-01	2.0E-01	2.1E+01	9.1E+00	2.5E+00	1.1E+00	1.9E-01	8.0E-02	2.5E+01	1.1E+01
	Krakrama	4.6E-03	2.0E-03	1.1E-01	4.7E-02	1.7E+01	7.4E+00	3.2E-01	1.4E-01	4.4E-01	1.9E-01	1.8E+01	7.7E+00
	Bonny	3.6E-03	1.6E-03	1.9E-01	8.2E-02	7.6E+00	3.3E+00	1.9E-01	8.2E-02	6.0E-01	2.6E-01	8.6E+00	3.7E+00
	Mean THQ	6.1E-02	2.6E-02	3.4E-01	1.5E-01	1.8E+01	7.8E+00	1.5E+00	6.5E-01	3.6E-01	1.5E-01	2.0E+01	8.8E+00
												**TCR = ∑CR**	
*P. monodon*	Buguma	7.6E-06	1.6E-05	1.8E-04	3.9E-04	5.0E-04	1.1E-03	1.0E-05	2.2E-05	1.2E-03	2.6E-03	1.9E-03	4.1E-03
	Krakrama	2.4E-07	5.1E-07	1.1E-05	2.3E-05	6.1E-04	1.3E-03	4.7E-06	1.0E-05	1.9E-03	4.0E-03	2.5E-03	5.3E-03
	Bonny	6.0E-08	1.3E-07	1.8E-05	3.8E-05	4.2E-04	8.9E-04	3.1E-06	6.6E-06	1.3E-03	2.7E-03	1.7E-03	3.7E-03
*T. fuscatus*	Buguma	5.4E-06	1.2E-05	6.8E-05	1.5E-04	2.2E-04	4.6E-04	5.3E-06	1.1E-05	5.8E-04	1.2E-03	8.7E-04	1.9E-03
	Krakrama	3.5E-06	7.6E-06	2.5E-06	5.4E-06	1.1E-04	2.3E-04	2.2E-06	4.8E-06	9.4E-04	2.0E-03	1.1E-03	2.3E-03
	Bonny	2.0E-07	4.4E-07	7.6E-06	1.6E-05	9.2E-05	2.0E-04	3.8E-06	8.2E-06	1.6E-03	3.5E-03	1.7E-03	3.7E-03
*C. rhizophorae*	Buguma	9.7E-06	2.1E-05	5.7E-05	1.2E-04	3.5E-04	7.5E-04	7.1E-06	1.5E-05	7.0E-04	1.5E-03	1.1E-03	2.4E-03
	Krakrama	2.3E-07	4.9E-07	1.4E-05	2.9E-05	2.8E-04	6.1E-04	9.1E-07	1.9E-06	1.6E-03	3.5E-03	1.9E-03	4.1E-03
	Bonny	1.8E-07	3.9E-07	2.4E-05	5.1E-05	1.3E-04	2.7E-04	5.3E-07	1.1E-06	2.2E-03	4.8E-03	2.4E-03	5.1E-03
	Mean THQ	3.0E-06	6.4E-06	4.3E-05	9.2E-05	3.0E-04	6.4E-04	4.2E-06	9.0E-06	1.3E-03	2.9E-03	1.7E-03	3.6E-03
